# Children's Country Holidays Fund

**Published:** 1889-08-10

**Authors:** 


### CHILDREN'S COUNTRY HOLIDAYS FUND.

Sending children for a fortnight's holiday in the country arouses the sympathy of everybody, and so popular has this form of charity become, that little funds are being started in every part of London.

Indeed, it seems likely that the charitable public are to be harassed in yet another field by rival societies, each touting for the custom of the benevolent.

The reasons for concerted action ought to be carefully considered, and the dangers of unorganised effort fairly faced by those who now seem to consider some slight incompatibility of temper a sufficient reason for separation.

In the case of the Children's Country Holidays Fund, it cannot be truly said that organisation has meant over-centralisation and office expenses. Many of the general expenses, which altogether amount to about five per cent. of the whole, would be incurred without any central committee or office, and the cost per head is actually greater in some (perhaps in most) of the ostentatiously "local" funds.

When a number of isolated committees federated themselves under a central council in 1884 they did so that they might with increased ease and efficiency in working secure a greater equality in the distribution of the public benevolence.

It is evident that a committee making grants from a central fund in proportion to the population and relative needs of all the different parts of London secure a far fairer division of benefits than is possible when the holiday depends on the eloquence of the vicar or the acquisition of a rich body of district visitors.

The council of the Children's Country Holidays Fund makes no distinction of creed or school.

On the question of increased ease and efficiency many people will at once join issue—they would rather incur the dangers of overlapping than admit that a big society can work successfully. Some object to filling up forms, but these need not mean formalism, but only order and so much uniformity as ensures fairness. If it is advisable to learn certain particulars as to earnings, so as to make possible a better selection of the most needy and to fix more accurately the amount each parent can contribute, it is obviously easier to have a form with printed columns. Similarly, it is necessary to send particulars of age and sex to the country visitor, and a form enables these to be placed in intelligible order.

But apart from forms, it is much easier to work in co-operation with others than alone. The experience of others must be an assistance, and comparison is always a spur to more careful method.

This is shown indirectly by the fact that the parents pay a far larger proportion of the cost in the large society than in any of the numerous private ventures. The very same people collect the money in both cases—*i.e.*, clergy, district visitors, school managers, etc.—and the children are taken from the same class.

The Children's Country Holidays Fund may fearlessly compare their Whitechapel children with those sent privately from portions of Marylebone with much lower parents' payments; while striking instances may be quoted of the same children going one year with a fair and cheerfully-given contribution from their parents, and in the next being sent free or at much lower rate through some smaller agency. Such a system must create jealousies and a tendency on the part of the poor to wait till the last moment on the chance of this pauperising gift.

There is no doubt a very great advantage derived from a close bond of sympathy between the visitor and the children, and this the Children's Country Holidays Fund tries to retain. Each of the thirty-six local committees are aggregates of individuals who are in personal contact with the children. The committee serves as a centre of experience and a source of the encouragement derived from the feeling of co-operation, but it in no way destroys personal sympathy.

The central council strives to give the local committees a free individual development, and concerns itself chiefly with the work of raising funds and seeing that when raised they are apportioned as fairly as possible among the different districts.

In fact the Children's Country Holidays Fund tries with the greater fairness and influence of a comprehensive organisation to combine all that is most valuable in individual work. It may not do everything perfectly, but it tries.

